# OCT4 promotes lung cancer progression through upregulation of VEGF-correlated chemokine-1

**DOI:** 10.7150/ijms.102505

**Published:** 2025-01-13

**Authors:** Bing-Hua Su, Chung-Teng Wang, Jia-Ming Chang, Huan-Yun Chen, Tang-Hsiu Huang, Yi-Ting Yen, Yau-Lin Tseng, Meng-Ya Chang, Che-Hsin Lee, Li-Hsin Cheng, Yu-Chih Wu, Chao-Liang Wu, Pin Ling, Ai-Li Shiau

**Affiliations:** 1 School of Respiratory Therapy, College of Medicine, Taipei Medical University, Taipei, Taiwan.; 2 TMU Research Center of Thoracic Medicine, Taipei Medical University, Taipei, Taiwan.; 3 Tong Yuan Diabetes Center, College of Medicine, National Cheng Kung University, Tainan 70101, Taiwan.; 4 Department of Microbiology and Immunology, College of Medicine, National Cheng Kung University, Tainan 70101, Taiwan.; 5 Thoracic Division, Department of Surgery, Ditmanson Medical Foundation Chiayi Christian Hospital, Chiayi 60002, Taiwan.; 6 Institute of Molecular Biology, National Chung Cheng University, Chiayi 62102, Taiwan.; 7 Division of Chest Medicine, Department of Internal Medicine, National Cheng Kung University Hospital, College of Medicine, National Cheng Kung University, Tainan 70101, Taiwan.; 8 Division of Thoracic Surgery, Department of Surgery, National Cheng Kung University Hospital, College of Medicine, National Cheng Kung University, Tainan 70101, Taiwan.; 9 Institute of Medical Sciences, Tzu Chi University, Hualien 97004, Taiwan.; 10 Department of Biological Sciences, National Sun Yat-sen University, Kaoshiung 80424, Taiwan.; 11 Core Laboratory of Organoids Technology, Office of R&D, Taipei Medical University, Taiwan.; 12 Department of Biochemistry and Molecular Biology, College of Medicine, National Cheng Kung University, Tainan 70101, Taiwan.; 13 Department of Medical Research, Ditmanson Medical Foundation Chiayi Christian Hospital, Chiayi 60002, Taiwan.; These authors contributed equally to this work.

**Keywords:** OCT4, VEGF-correlated chemokine-1, VCC-1, CXCL17, TGF-β, macrophage migration, lung cancer

## Abstract

Embryonic development and tumor genesis share numerous similarities, with OCT4 standing out as a pivotal transcription factor in embryonic development. Expression of OCT4 is associated with poor prognosis of lung adenocarcinoma. VEGF-correlated chemokine-1 (VCC-1), also known as C-X-C motif chemokine ligand 17 (CXCL17), has been suggested to play a role in promoting tumor angiogenesis and metastasis. In the present study, we show a positive correlation between OCT4 expression levels and tumor metastatic potential, where an increase in OCT4 expression parallels an upregulation of VCC-1 in lung cancer. This relationship was substantiated through DNA microarray analysis and further confirmed by tissue staining of clinical lung cancer samples, demonstrating a positive correlation between OCT4 and VCC-1 expression. In A549 and H1299 human lung cancer cells, modulations in OCT4 expression directly influenced VCC-1 levels, as evidenced by the reporter assay of the VCC-1 promoter, indicating the regulatory role of OCT4 in transactivating VCC-1 expression. Furthermore, enhanced VCC-1 expression in H1299 cells promoted transforming growth factor-β (TGF-β) secretion, contributing to lung cancer cell aggressiveness. Additionally, VCC-1 secretion by H1299 cells could attract THP-1 macrophages, further implicating its role in tumor progression. NOD/SCID mice inoculated with VCC-1-knockdown A549 lung cancer cells exhibited significantly smaller tumors than those inoculated with control cells. On the basis of these findings, we highlight the importance of the OCT4-VCC-1 axis in lung cancer progression. Our findings also provide therapeutic targets for lung cancer.

## Introduction

Lung cancer is divided into two main types: small cell lung carcinoma (SCLC) and non-small cell lung carcinoma (NSCLC). SCLC, comprising 15% of cases, proliferates and spreads early, usually treated with chemotherapy or radiotherapy. NSCLC, accounting for 80-85% of cases, grows slower and often spreads later in the disease. It includes squamous cell carcinoma, adenocarcinoma, and large cell carcinoma, each with specific characteristics. Surgery is common in early-stage NSCLC, while chemotherapy or radiotherapy is used if cancer has spread. The difficulty in early diagnosis contributes to high mortality rates, with late discovery leading to metastasis and poor prognosis, resulting in a 5-year survival rate of only 15%. Identifying abnormal gene expression in early-stage lung cancer for diagnosis and prognosis has become a crucial focus of recent research 1.

Tumors share characteristics with early embryonic cells, leading researchers to explore embryonic gene expression for cancer treatment. Homeodomain proteins (HD), which are important in embryonic development, can become dysregulated, contributing to various cancers 2. OCT4, a homeodomain protein, is overexpressed in tumors, correlating with aggressive growth 3-5. OCT4 regulates genes involved in cell differentiation and maintenance of pluripotency. Its expression can indicate cancer presence and prognosis 6. Known downstream genes regulated by OCT4 include Fgf4, Utf1, Spp1/Opn, Fbxo15/Fbx15, Sox2, Pdgfα/PDGFα, Cga/α, βhCG, Ifngr1/γIFN, and Zfp42/Rex1 7-12. The functions of OCT4 and cancer stem cells in the development of human tumors and metastasis have been extensively investigated. We have previously reported that OCT4 expression in bladder cancer is predictive of tumor progression and metastasis 6. Moreover, targeting of OCT4 represents a viable strategy for the treatment of primary and metastatic cancers that overexpress OCT4 13, 14. In lung cancer, our research revealed that OCT4 plays a role in M2 macrophage polarization by upregulating macrophage colony-stimulating factor (M-CSF), which in turn contributes to cancer growth and metastasis 4. It is noteworthy that our previous microarray analysis revealed a 7-fold increase in the expression of VEGF-correlated chemokine-1 (VCC-1) in bladder cancer cells with high OCT4 expression 6, suggesting a potentially significant correlation between VCC-1 and OCT4 in the progression of tumorigenesis. Nevertheless, further research is required to elucidate the mechanisms by which OCT4 regulates VCC-1 and their correlation in lung cancer.

VCC-1, also known as C-X-C motif chemokine ligand 17 (CXCL17) or dendritic cell and monocyte chemokine-like protein (DMC), is minimally expressed in the lung and trachea. Microarray analysis has shown a positive correlation between VCC-1 and VEGF expression, suggesting a role for VCC-1 in promoting tumor angiogenesis and metastasis 15. Immunological studies indicate that VCC-1 attracts dendritic cells and monocytes, and its structure predicted from its nucleotide sequence is similar to IL-8, suggesting functional similarities to CXCL8 and CXCL14 16. VCC-1, which is highly expressed in colon, breast, and gastric cancer, promotes the invasion of tumor cells 17-19. These findings suggest that VCC-1 expression is associated with tumor progression, making it a potential diagnostic and therapeutic marker for cancer. Nevertheless, additional research is required to gain a comprehensive understanding of its molecular mechanisms and functions in lung cancer.

In this study, we demonstrate a positive correlation of OCT4 and VCC-1 expression with the stage severity of clinical lung cancer. Increased OCT4 expression was associated with elevated levels of VCC-1. In human lung cancer cell lines, OCT4 overexpression in OCT4-low-expressing H1299 cells increased, whereas OCT4 knockdown in OCT4-high-expressing A549 cells decreased, VCC-1 expression and transforming growth factor-β (TGF-β) secretion. Overexpression of OCT4 or VCC-1 in H1299 cells attracted the migration of macrophage-like THP-1 cells, contributing to tumor aggressiveness. Furthermore, knockdown of VCC-1 in A549 cells decreased THP-1 cell migration* in vitro* and reduced tumor growth in NOD/SCID mice, highlighting the role of VCC-1 in tumor growth. Collectively, our results indicate that VCC-1 is a downstream regulator of OCT4 and is a promising biomarker and therapeutic target for lung cancer, emphasizing the critical roles of OCT4 and VCC-1 in cancer development.

## Materials and Methods

### Cell lines and mice

Human embryonic lung cells (HEL299), non-small cell lung carcinoma cells (H1299), lung adenocarcinoma epithelial cells (A549), and human acute monocytic leukemia cells (THP-1), which were originally obtained from Bioresource Collection and Research Center, Taiwan, were cultured in Dulbecco's modified Eagle's medium (DMEM) supplemented with 2 mm L-glutamine, 10% cosmic calf serum (Hyclone, Logan, UT, USA), and 50 µg/ml gentamicin. A panel of human lung adenocarcinoma cell lines (CL1-0, CL1-1, CL1-3, CL1-5, and CL1-5F4), harbored mutated p53 with gradually increasing invasiveness, was generously provided by Dr. Pan-Chyr Yang 20. The CL1 cell line was originally derived from a 64-year-old man suffering from poorly differentiated lung adenocarcinoma. Human lung adenocarcinoma cell lines were cultured in minimum essential medium supplemented with 2 mM L-glutamine, 10% cosmic calf serum, and 50 µg/ml gentamicin. NOD/SCID mice at 6-8 weeks of age were purchased from the Laboratory Animal Center of National Cheng Kung University (NCKU). The experimental protocol adhered to the rules of the Animal Protection Act of Taiwan and was approved by the Laboratory Animal Care and Use Committee of NCKU. Lung clinical specimens from patients with different stages of lung cancer (Grade I-III) were obtained from the Department of Thoracic Surgery at NCKU Hospital (IRB number: B-ER-105-406).

### Construction of OCT4 and VCC-1 expression vectors and generation of lentiviral vectors encoding OCT4 or VCC-1 shRNAs

The lentiviral vector encoding human OCT4 (pSin-EF2-OCT4-Pur, Addgene plasmid 16579) was obtained from Addgene (http://www.addgene.org). Furthermore, the GFP cDNA fragment was excised from pEGFP-N1 (Clonetech, Palo Alto, CA, USA) by digestion with *Nhe*I and *Mfe*I and then cloned into the *Spe*I/*Eco*RI sites of pSin-EF2-OCT4-Pur to replace OCT4 and generate a control lentiviral vector encoding GFP, designated pSin-EF2-GFP-Pur. In addition, the cDNA fragment of human *OCT4* was obtained from pSin-EF2-OCT4-Pur by polymerase chain reaction (PCR) amplification using *OCT4*-specific primers (forward: 5'-GTCCGAGTGTGGTTCTGTA-3' and reverse: 5'-CTCAAGTTTGAATGCATGGGA-3') and cloned into a TA vector (yT&A cloning vector kit, Yeastern Biotech, Taipei, Taiwan). The PCR amplicons were subsequently subjected to digestion with *Bgl*II and *Sal*I, followed by subcloning into the *Bam*HI/*Sal*I sites of pCMV-Tag2B (Stratagene, La Jolla, CA, USA), resulting in the generation of a Flag-tagged OCT4 expression plasmid (pCMV-Tag2B-OCT4). To synthesize human *VCC-1* cDNA, total RNA was isolated from A549 cells using a QuickGene RNA Cultured Cell Kit (AutoGene, Holliston, MA, USA) and used for cDNA synthesis using PrimeScript™ High Fidelity RT-PCR Kit (Takara Bio, Otsu, Japan) according to the manufacturer's instruction. The human *VCC-1* cDNA was amplified using specific primers (forward: 5'- ATGAAAGTTCTAATCTCTTCCCTC-3' and reverse: 5'-CTACAAAGGCAGAGGCAAAGCTTC-3') and subsequently cloned into the TA vector to generate yT&A-hVCC-1, which was further subcloned into pCMV-Tag2B to produce pCMV-Tag2B-VCC-1.

Lentiviruses carrying OCT4 or VCC-1 shRNA were generated utilizing the packaging plasmid (psPAX2), envelope plasmid (pMD2.G), and OCT4- or VCC-1-specific shRNA obtained from the National RNAi Core (Academia Sinica, Taiwan). For OCT4 or VCC-1 knockdown experiments, pLKO.1-puro-based lentiviral vectors including stem-loop cassettes encoding shRNA specific to human *OCT4* (TRCN0000004880 and TRCN0000004883; designated OCT4 shRNA 1 and 2), human *VCC-1* (TRCN0000134061, TRCN0000138165, TRCN0000137946, TRCN0000136414, TRCN0000136148; designated VCC-1 shRNA 1-5), and luciferase (TRCN0000072246; designated Luc shRNA) were used. Prior to lentivirus production, 293T cells were evenly seeded on a 10-cm cell culture dish. Transfection was performed the next day using a calcium phosphate precipitation method. Following 16-h incubation, the culture medium was replenished with fresh medium and collected after 48 h for storage at -20° C for future use. Various recombinant lentiviruses encoding OCT4 or VCC-1 shRNAs were produced as previously described 21.

For stable overexpression of OCT4 in lung cancer cells, H1299 cells were transfected with 4 µg of pSin-EF2-OCT4-Pur or pSin-EF2-GFP-Pur, or mock-transfected. In addition, to study dose-response effects of OCT4, we transfected H1299 cells with various amounts of pSin-EF2-OCT4-Pur or pCMV-Tag2B-OCT4 and the control plasmid pSin-EF2-GFP-Pur or pCMV-Tag2B. The total amount of plasmid DNA for transfection was kept constant by adding the control vector. Same procedures were performed for stable overexpression of VCC-1 in H1299 cells except that pCMV-Tag2B-VCC-1 was used for transfection.

### Immunoblotting and immunohistochemistry

For immunoblotting, 30 μg of cell lysates were subjected to 10% SDS-polyacrylamide gel electrophoresis and subsequently transferred to PVDF membranes (Millipore, Bedford, MA, USA). Subsequently, the membranes were incubated with mouse monoclonal anti-OCT4 antibody (GT486, No. GTX627419, Genetex, Hsinchu, Taiwan) or rabbit anti-VCC-1 antibody (#18108-1-AP, Proteintech, Chicago, IL, USA). Antibody-protein complexes were detected with horseradish peroxidase (HRP)-conjugated goat anti-rabbit IgG (1:10,000, Jackson Immuno Research, West Grove, PA, USA) and visualized using an Immobilon Western Chemiluminescent HRP Substrate (Merck Millipore, Burlington, MA, USA). Subsequently, β-actin was detected with mouse monoclonal anti-β-actin-peroxidase antibody (A3854, Sigma-Aldrich, St. Louis, MO, USA) on the same membrane to serve as a loading control. For immunohistochemical analysis, the paraffin was removed from the paraffin-embedded tissue sections using xylene, followed by rehydration. The sections were then incubated with protease K for 10 min to facilitate antigen retrieval, followed by a 10-min incubation with 10% hydrogen peroxide to block endogenous peroxidase activity. Following blockade with a buffer containing 10% bovine serum albumin, slides were incubated with mouse monoclonal anti-OCT4 (Genetex) or rabbit anti-VCC-1 antibody (Proteintech) for 12 h. Subsequently, appropriate HRP-labeled secondary antibodies and 3-amino-9-ethylcarbazole (Thermo Fisher Scientific, Waltham, MA, USA) were used as the substrate chromogen, and the slides were counterstained with hematoxylin.

### Real-time quantitative RT-PCR (qPCR)

Total RNA was isolated from plasmid-transfected or lentivirus-transduced cells using a QuickGene RNA Cultured Cell Kit (AutoGene, and RNA was used for cDNA synthesis using PrimeScript™ RT Reagent Kit (Takara Bio) according to the manufacturer's instruction. The following primers were used for qPCR: human *OCT4* (*POU5F1*), 5'-CCTGAAGCAGAAGAGGATCACC-3' (forward) and 5'-AAAGCGGCAGATGGTCGTTTGG-3' (reverse); human *VCC-1* (*CXCL17*), 5'-ACAGTGTCTGGGCTGCCAAAGA-3' (forward) and 5'-GGCTCTGGAATGCTTGTTTGGC-3' (reverse); human *GAPDH*, 5'-ACTTCAACAGCGACACCCACT-3' (forward) and 5'-GCCAAATTCGTTGTCATACCAG-3' (reverse). The cDNA and primers were mixed with the SYBR premix Ex Taq (Takara Bio). The relative mRNA expression of different genes was determined using the 2^-ΔΔCT^ method, with the value obtained by subtracting the Ct value for GAPDH mRNA from the Ct value for different mRNA species.

### Reporter plasmid construction and luciferase assay

The 2,000-bp human *VCC-1* promoter region upstream of the transcription start site was PCR amplified using the primer pair (forward: 5'-TGACACAAATAATGTTCTTGAGA-3' and reverse: 5'-GCTTTAGTCCCAGGCCAGCGTTC-3') with genomic DNA from human 293T cells as the template. The PCR fragment was cloned into the TA vector to generate yT&A-VCC-1 plasmid. The plasmid DNA was then digested with *Eco*RI and *Sal*I, and the released *VCC-1* promoter fragment was cloned into a dual-luciferase reporter vector pFRL2 22 that had been digested with *Eco*RI and *Xho*I, resulting in pFRL2-VCC-1p. H1299 cells were transfected with 4 μg of pFRL2-VCC-1p using PEI for 24 h. Cell lysates were harvested 48 h after transfection, and their firefly and *Renilla* luciferase activities were determined using a Dual-Luciferase® Reporter Assay System (Promega, Madison, WI, USA) to measure the *VCC-1* promoter activity. The ratio of firefly luciferase activity to *Renilla* luciferase activity was expressed as relative light units (RLU).

### Chromatin immunoprecipitation (ChIP) assay

ChIP was performed using the EZ-ChIP kit (Merck Millipore) according to the manufacturer's instructions. A549 cells (1 × 10^7^) grown in 10-cm culture dishes were cross-linked with 1% formaldehyde for 10 min at 37° C. After cross-linking, the cells were washed with PBS, harvested in 2 ml PBS, and pelleted by centrifugation. The cell pellets were then resuspended in 1 ml SDS lysis buffer containing a protease inhibitor cocktail from the ChIP kit. The lysates were sonicated to shear the chromatin into fragments averaging approximately 500 to 1,000 bp in length. After sonication, the samples were centrifuged, and the supernatants were diluted with the ChIP kit dilution buffer. Each sample was mixed with the dilution buffer and protein G agarose beads, then rotated at 4° C for 1 h to pre-clear the chromatin. Following centrifugation, the supernatants were transferred to new tubes. For immunoprecipitation, the chromatin solution was incubated with mouse monoclonal anti-human OCT4 antibody (sc-5279, Santa Cruz Biotechnology, Santa Cruz, CA, USA) overnight at 4° C on a rotator. As a negative control, the chromatin solution was immunoprecipitated with purified normal mouse IgG. After immunoprecipitation, the samples were washed, and the chromatin was eluted. The cross-links were reversed by incubating the samples with 5 M NaCl at 65° C for 6 h. Finally, the DNA was purified and used for PCR to amplify the *VCC-1* promoter region containing the OCT4 binding sites. The PCR primers used in ChIP were 5'-ACTGCACCCATATCTCTAGGCT-3' (forward) and 5'-CCTGTTGACTTGTCACAGAGTTG -3' (reverse). The PCR products were separated by 1% agarose gel electrophoresis.

### Enzyme-linked immunosorbent assay (ELISA)

Levels of TGF-β and VEGF in the culture medium of H1299 and A549 cells under different conditions were quantified by a solid-phase sandwich ELISA (DuoSet ELISA kits, R&D, Minneapolis, MN, USA), according to the manufacturer's instructions. Briefly, 96-well microplates were coated with TGF-β or VEGF capture antibodies and incubated overnight at room temperature. Plates were then washed with PBST (0.05% Tween 20 in PBS) and blocked with 1% BSA in PBS at room temperature for 2 h. After additional washes, either standards or samples were added to each well and incubated at room temperature for 2 h. Following further washes, TGF-β or VEGF detection antibodies were added and incubated for 2 h, followed by incubation with Streptavidin-HRP for 20 min. The colorimetric reaction was initiated by adding tetramethylbenzidine/H_2_O_2_ substrate and terminated with 2 N H_2_SO_4_. The absorbance was measured at 450 nm and 590 nm using an ELISA reader to quantify VEGF and TGF-β levels.

### Differentiation of monocytic THP-1 cells into macrophage-like cells and cell migration assay

Human acute monocytic leukemia THP-1 cells were induced to differentiate into macrophage-like cells by treatment with 5 ng/ml phorbol 12-myristate 13-acetate (PMA) for 48 h. These cells were further treated with 5 nM human VCC-1 recombinant protein or 90 ng/ml human IL-4 to enhance differentiation for 24 h. Cell migration was analyzed using Boyden chamber assays with 8-μm pore polycarbonate filters (Neuro Probe, Gaithersburg, MD, USA) coated with 0.1 μg/ml of gelatin (Sigma-Aldrich). Various derivatives of H1299 cells (5 × 10^5^/well), including H1299/Vector, H1299/OCT4, and H1299/VCC-1, or A549/shVCC-1 and A549/shLuc cells, were seeded in the lower chambers for 24 h. The lower chambers containing cultured medium were covered with the filters. PMA-induced THP-1 cells were incubated with serum-free RPMI medium for 4 h and then transferred to the upper chamber. Following a 4-h incubation at 37° C, the cells were fixed with methanol and stained with Giemsa solution for 1 h. The cells located on the upper surface of the filter were then scraped off with cotton buds. The migrated cells on the underside of the filter were subsequently photographed and counted using phase-contrast microscopy.

### Cell proliferation assay

Cell proliferation was examined using a Cell Counting Kit-8 (CCK-8) assay (Dojindo Laboratories, Kumamoto, Japan). A549/shVCC-1, A549/shLuc, and parental A549 cells were seeded in 96-well plates and incubated for 24 h. CCK-8 reagent was added directly to each well, mixed by gentle tapping, and incubated for 1-4 h until an orange color developed. Absorbance was measured at 450 nm to assess cell viability.

### Animal studies

A549 cells were transduced with lentiviral vectors encoding VCC-1 shRNAs (shVCC-1-1 and shVCC-1-2) to knockdown VCC-1, followed by selection with 2 μg/ml puromycin to isolate stable clones. Groups of NOD/SCID mice were subcutaneously inoculated on the back with 1 × 10^6^ shVCC-1, shLuc, or parental A549 cells. Palpable tumors were measured at different time points in two perpendicular axes with a tissue caliper, and the tumor volume was calculated using the formula: (length of tumor) × (width of tumor)^2^ × 0.45. The mean tumor volumes were calculated when all the mice within the same group were alive. All animal experimental protocols were approved by the Animal Care and Use Committee of NCKU (IACUC approval number: 98023), and all experiments were performed following relevant regulations and guidelines.

### Statistical analysis

Data are expressed as mean ± SEM. Statistical significance between groups was assessed using one-way ANOVA with *p* values less than 0.05 considered statistically significant. Tumor volumes were compared using two-way ANOVA. Any *p*-value less than 0.05 was regarded as statistically significant.

The sources of all reagents were additionally provided in Supplementary Table: A list of reagents and resources used in the study.

## Results

### Expression of OCT4 and VCC-1 is elevated in clinical lung adenocarcinoma specimens and shows a positive correlation

**Figure 1:**
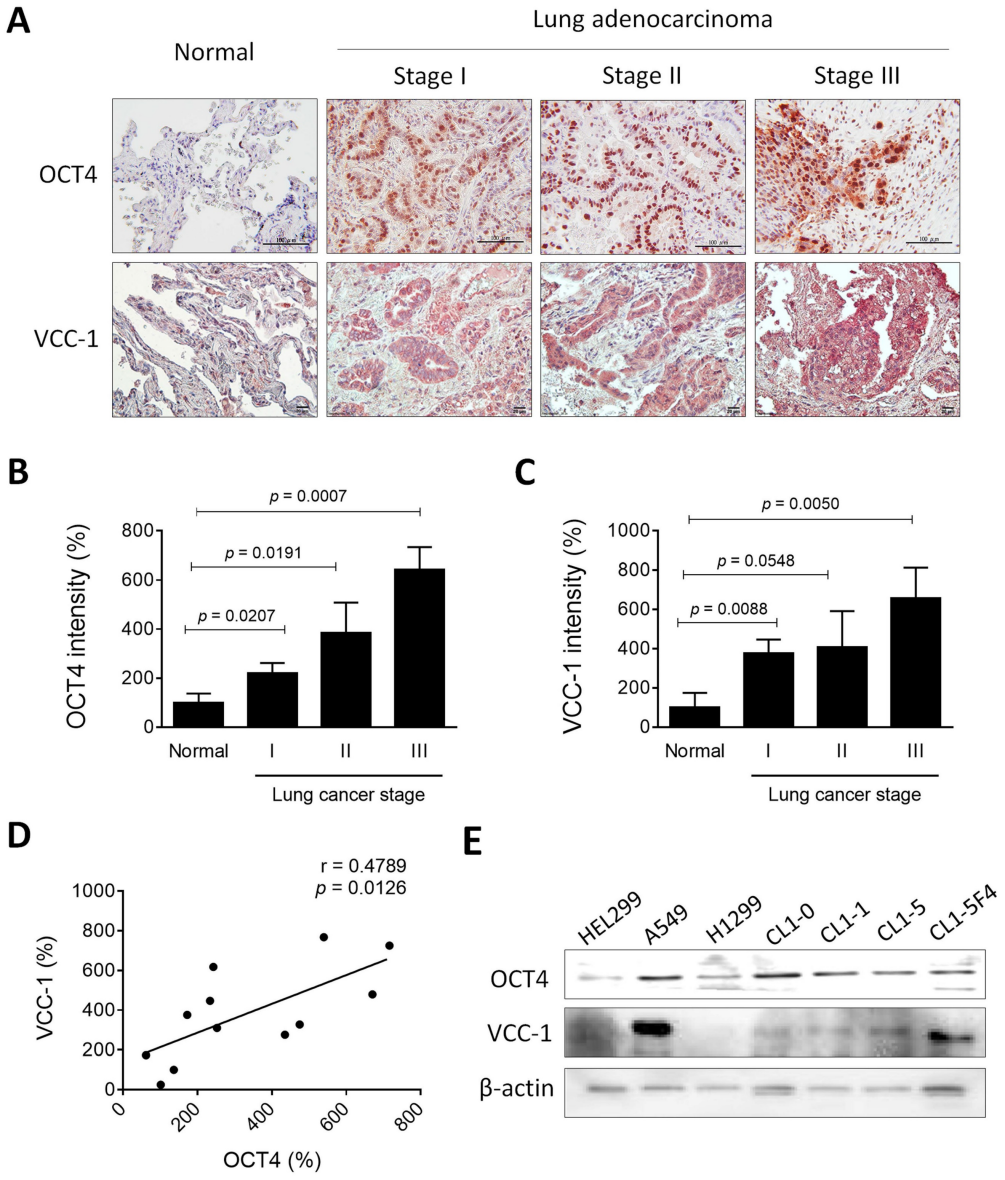
** Expression of OCT4 and VCC-1 in clinical lung adenocarcinoma tissues and lung cancer cell lines. (A-C)** Immunohistochemical staining (A) and quantification (B, C) of OCT4 and VCC-1 expression in lung adenocarcinoma patients (Stage I, n = 3; Stage II, n = 3; Stage III, n = 3) and normal lung tissues (Normal), (Scale bars, 100 μm for OCT4; 20 μm for VCC-1). (**D**) Positive correlation between OCT4 and VCC-1 intensities (r = 0.4789, *p* = 0.0126, Pearson's correlation coefficient). **(E)** Expression of OCT4 and VCC-1 in six lung cancer cell lines and HEL299 normal lung fibroblasts detected by immunoblot analysis. Expression of β-actin served as the loading control.

As a transcription factor, OCT4 regulates the expression of downstream genes through its binding to the OCT4 response element (ORE) 23. We previously employed the microarray analysis to investigate potential OCT4 downstream genes in cancer cells. We found that, in cells where OCT4 was highly expressed, there was a marked 7-fold increase in VCC-1 expression 6. To verify whether the expression patterns of OCT4 and VCC-1 were positively correlated in lung cancer, we assessed the expression of OCT4 and VCC-1 in nine human primary lung adenocarcinoma specimens with varying stages collected from National Cheng Kung University (NCKU) Hospital. Notably, immunohistochemical staining revealed that tumor tissues exhibited elevated OCT4 and VCC-1 expression relative to normal tissues (Figure 1A). With regard to the immunointensity of OCT4 and VCC-1, expression levels of OCT4 (Figure 1B) or VCC-1 (Figure 1C) were positively correlated with the stage severity of lung adenocarcinoma. Furthermore, a positive correlation was observed between the expression of OCT4 and VCC-1 (r = 0.4789, *p* = 0.0126) (Figure 1D), suggesting a potential association between VCC-1 and OCT4 expression in lung adenocarcinoma.

OCT4 exhibits variable expression across lung cancer cells and, to a lesser extent, in immortalized lung fibroblasts. Overall, elevated levels were observed in various lung cancer cell lines (Figure 1E). Among the lung cancer cell lines tested, A549 cells exhibited higher endogenous OCT4 expression than H1299 cells (Figure 1E). Furthermore, VCC-1 expression was observed in all tested lung cancer cell lines, whereas its expression was minimal in immortalized HEL299 lung fibroblasts (Figure 1E). Of note, detection of VCC-1 expression in an established series of human lung cancer cell lines with varying invasive and metastatic activities (CL1-0, CL1-1, CL1-5, and CL1-5-F4) revealed a positive correlation between VCC-1 expression and cancer invasiveness, suggesting a close association between VCC-1 expression levels and tumor progression (Figure 1E). These results collectively suggest that OCT4 and VCC-1 represent novel tumor biological and prognostic markers for lung cancer. Moreover, the expression of OCT4 in human primary lung cancer may induce VCC-1 expression, which may contribute to tumor progression.

### OCT4 functions as an upstream regulator of VCC-1 expression

**Figure 2:**
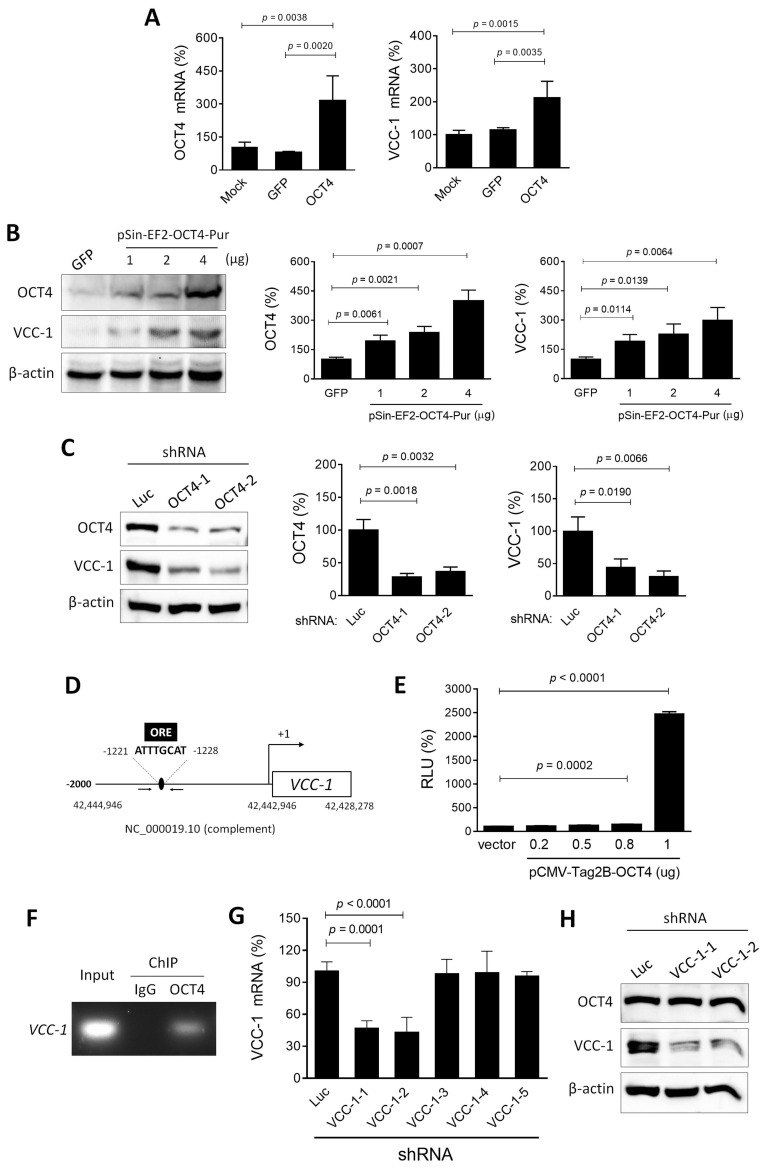
** Overexpression of OCT4 increases, whereas knockdown of OCT4 decreases, VCC-1 expression in lung cancer cells.** (**A-C**) Detection of OCT4 and VCC-1 expression. H1299 cells were transfected with 4 µg of pSin-EF2-OCT4-Pur (OCT4) or pSin-EF2-GFP-Pur (GFP), or mock-transfected (A), or with 1, 2, and 4 µg of pSin-EF2-OCT4-Pur or pSin-EF2-GFP-Pur (4 µg) (B), or transduced with lentiviral vectors expressing shRNAs specific to OCT4 (#1 or #2) or luciferase (Luc) (C). After 48 h, levels of OCT4 and VCC-1 mRNA were examined by qPCR (A, n = 4), and their protein levels were detected by immunoblotting (B, C, n = 3). Expression of β-actin served as the loading control. Representative immunoblots from three independent experiments (left panels) and quantitative analysis of OCT4 (middle panels) and VCC-1 (right panels) are shown. Ratios between the intensity of the bands corresponding to the indicated protein and those corresponding to β-actin were calculated, and ratios of control cells were arbitrarily set to 100. (**D**) A schematic representation of an OCT4 binding site located between -1,220 bp and -1,228 bp relative to the transcription start site within the promoter of human *VCC-1* gene (NCBI Gene ID: 284340) located on chromosome 19 (NC_000019.10, position: complement 42,428,278-42,442,946). (**E**) Reporter assay for OCT4 transactivation activity. H1299 cells that had been transfected with pCMV-Tag2B (Vector) or pCMV-Tag2B-OCT4 (OCT4) were cotransfected with the pFRL2-VCC-1 reporter plasmid. Total cell lysates were harvested 48 h later, and their firefly and *Renilla* luciferase activities were determined by a dual-light luciferase reporter assay system. The ratio of firefly luciferase activity to *Renilla* luciferase activity was expressed as relative light units (RLU). Values shown are the mean ± SEM. (n = 3). (**F**) ChIP assay showing direct binding of OCT4 to the ORE of human *VCC-1* promoter. Cross-linked chromatin of A549 cells was immunoprecipitated with mouse anti-human OCT4 antibody or normal mouse IgG in combination with protein G agarose beads, followed by PCR amplification of the *VCC-1* promoter region encompassing OCT4 binding sites. (**G**) Knockdown efficiency of various VCC-1 shRNAs in A549 cells. Cells were transduced with lentiviral vectors expressing shRNAs specific to VCC-1 (#1 to #5) or luciferase (Luc) for 48 h. Levels of VCC-1 mRNA were examined by qPCR (n = 4). Note that VCC-1-1 and VCC-1-2 shRNAs displayed VCC-1-knockdown activities. (**H**) Detection of OCT4 and VCC-1 expression in VCC-1-knockdown A549 cells. Cells were transduced with lentiviral vectors expressing shRNAs specific to VCC-1 (#1 or #2) or luciferase (Luc) for 48 h, and their lysates were examined for OCT4 and VCC-1 expression by immunoblotting. Expression of β-actin served as the loading control.

Given the positive correlation between the expression of OCT4 and VCC-1 observed in lung adenocarcinoma (Figure 1), we proceeded to investigate the regulatory relationship between OCT4 and VCC-1. In lung cancer cell lines, A549 cells exhibited higher endogenous OCT4 expression than H1299 cells. Our subsequent studies employed OCT4 overexpression in H1299 cells and OCT4 knockdown in A549 cells. H1299 cells were transduced with lentiviral vectors encoding *OCT4* or *GFP*, or transfected with an OCT4 expression plasmid (pSin-EF2-OCT4) or a control plasmid (pSin-EF2-GFP). Thereafter, RT-PCR and immunoblotting analyses were performed to analyze the expression of OCT4 and VCC-1. As expected, H1299/OCT4 cells expressed higher levels of OCT4 mRNA, whereas OCT4 expression in H1299/GFP and parental (mock) cells was at similar levels (Figure 2A, left). Overexpression of OCT4 increased VCC-1 mRNA expression (Figure 2A, right). At protein levels, overexpression of OCT4 increased VCC-1 protein levels in a dose-response manner (Figure 2B). In contrast, transduction of A549 cells with lentiviral vectors carrying *OCT4* shRNA resulted in a corresponding decrease in VCC-1 protein levels in two knockdown clones (Figure 2C), suggesting that OCT4 may have a regulatory role in VCC-1 expression at the transcriptional level. We further validated that OCT4 could transactivate the *VCC-1* promoter. OCT4, a member of the POU family of transcription factors, recognizes and binds to the octamer motif (consensus sequence ATGCAAAT/ATTTGCAT) via its bipartite DNA-binding POU domain 24. The human *VCC-1* gene (NCBI Gene ID: 284340) is located on chromosome 19 (Homo sapiens chromosome 19, GRCh38.p14 Primary Assembly, NC_000019.10, position: complement 42,428,278-42,442,946). We identified an OCT4 binding site (ATTTGCAT) within the promoter region, positioned between -1,220 bp and -1,228 bp relative to the transcription start site (Figure 2D). Co-transfection of pFRL2-*VCC-1*p and pCMV-Tag2B-hOCT4 in H1299 cells revealed concomitant increases in OCT4 expression and *VCC-1* promoter activity, as examined by a luciferase reporter assay (Figure 2E). We further validated the involvement of the OCT binding site within the *VCC-1* promoter in OCT4-induced VCC-1 expression by a ChIP assay. Chromatin was extracted from A549 cells, sonicated, and immunoprecipitated with an anti-human OCT4 antibody or normal mouse IgG serving as a negative control. PCR amplification of the *VCC-1* promoter region produced a 225-bp fragment in the OCT4 immunoprecipitates, but not in the IgG control, confirming that OCT4 specifically binds to the *VCC-1* promoter (Figure 2F). Reciprocally, using VCC-1-knockdown A549 cells, we investigated whether VCC-1 could regulate OCT4 expression. Figure 2G show that two VCC-1 shRNAs (VCC-1-1 and VCC-1-2) among five tested shRNAs could significantly inhibit VCC-1 mRNA expression compared to the control shLuc. However, VCC-1 knockdown did not affect OCT4 expression (Figure 2H). Taken together, these results indicate that OCT4 transactivates VCC-1 expression by directly binding to the *VCC-1* promoter in lung cancer cells. Thus, VCC-1 is a downstream target of OCT4.

### Overexpression of VCC-1 in lung cancer cells increases TGF-β but not VEGF expression

**Figure 3:**
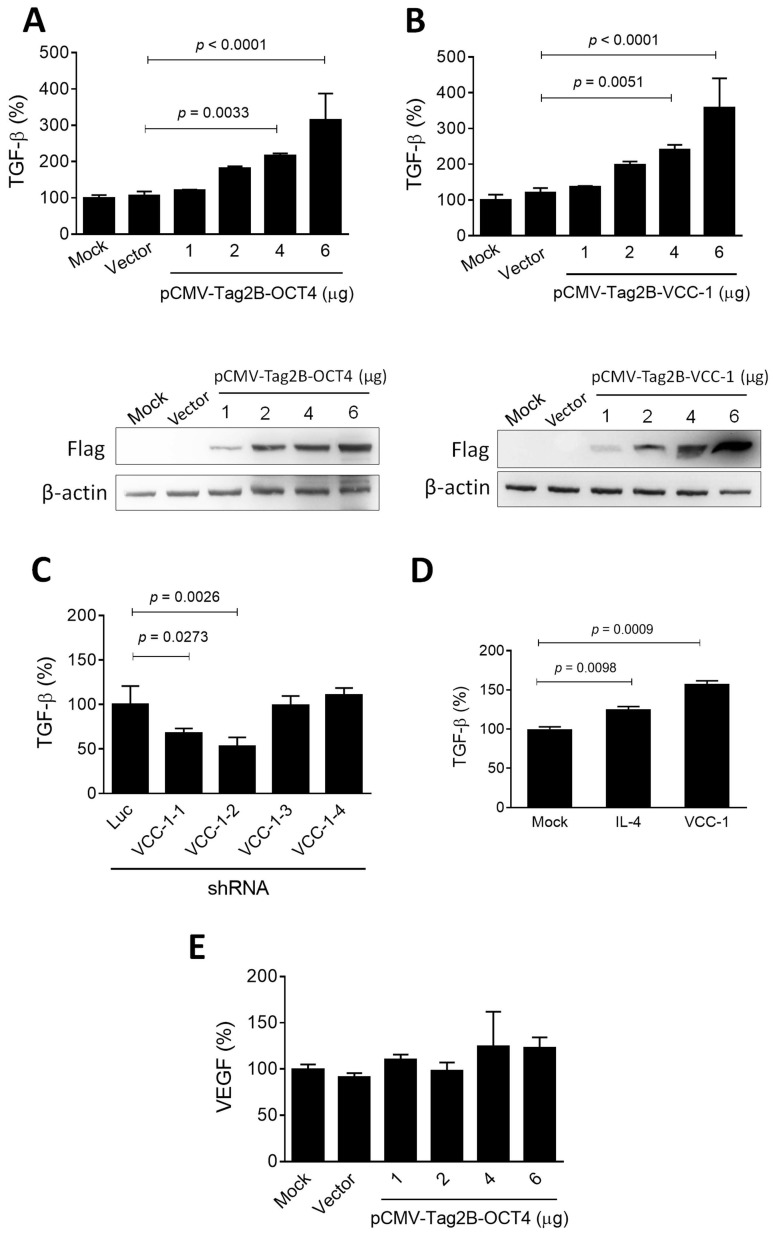
** OCT4 and VCC-1 promote TGF-β production in lung cancer cells.** (**A-C**) Detection of TGF-β production by ELISA. H1299 cells were transfected with pCMV-Tag2B (Vector, 6 µg), pCMV-Tag2B-OCT4 (OCT4, 1, 2, 4, and 6 µg), pCMV-Tag2B-VCC-1 (VCC-1, 1, 2, 4, and 6 µg), or mock-transfected (A, B). A549 cells were transduced with lentiviral vectors expressing shRNAs specific to VCC-1 (#1 to #4) or luciferase (Luc) (C) for 48 h. Dose-dependent overexpression of OCT and VCC-1 in H1299 cells transfected with Flag-tagged OCT4 and VCC-1 expression vectors were verified by immunoblotting with the anti-Flag antibody, respectively (lower panels, A, B). The culture medium was analyzed for TGF-β production by ELISA (upper panels, A-C, n = 3). (**D**) IL-4 and VCC-1 proteins enhance TGF-β production. THP-1 cells were treated with PMA (5 ng/ml) for 48 h, and stimulated with recombinant IL-4 (90 ng/ml) or VCC-1 (5 nM) proteins for 24 h. Levels of TGF-β in the culture medium were determined by ELISA at 48 h post-treatment (n = 3). (**E**) Detection of VEGF in H1299 cells that had been transfected with pCMV-Tag2B-OCT4 (OCT4), pCMV-Tag2B (Vector), or mock-transfected for 48 h. The culture medium was analyzed for VEGF production by ELISA (n = 3). Note that overexpression of OCT4 does not affect VEGF expression.

TGF-β is released and activated by malignant and non-cancer cells within the tumor microenvironment, where it exerts highly regulated and differential effects on multiple cell types, thereby promoting cancer progression 25. Given that OCT4 transactivates VCC-1 expression (Figure 2) and is associated with tumor progression (Figure 1) in human lung adenocarcinoma, we further investigated whether overexpression of OCT4 and VCC-1 have an impact on the regulation of TGF-β. Upon overexpression of OCT4 and VCC-1 in H1299 cells by transfection of pCMV-Tag2B-OCT4 and pCMV-Tag2B-VCC-1, respectively, a notable augmentation in TGF-β production was observed in cancer cells overexpressing OCT4 (Figure 3A) and VCC-1 (Figure 3B) in a dose-dependent manner, as examined by ELISA. Conversely, efficient knockdown of VCC-1 expression by VCC-1-1 and VCC-1-2 shRNAs reduced TGF-β production, whereas VCC-1-3 and VCC-1-4 shRNAs incapable of silencing VCC-1 expression did not alter TGF-β levels in A549 cells (Figure 3C). These results indicate the regulatory role of VCC-1 on TGF-β production in lung cancer cells. Moreover, both recombinant IL-4 and VCC-1 proteins could stimulate TGF-β production in THP-1 macrophages (Figure 3D). It is noteworthy that overexpression of OCT4 in H1299 cells had no notable effects on VEGF expression (Figure 3E), suggesting a lack of correlation between VCC-1 expression and VEGF in the context of lung cancer. Collectively, these results indicate that the elevated expression of VCC-1 in lung cancer cells can enhance TGF-β levels, which may promote tumor progression.

### Lung cancer cells overexpressing OCT4 or VCC-1 attract the migration of macrophage-like THP-1 cells

**Figure 4:**
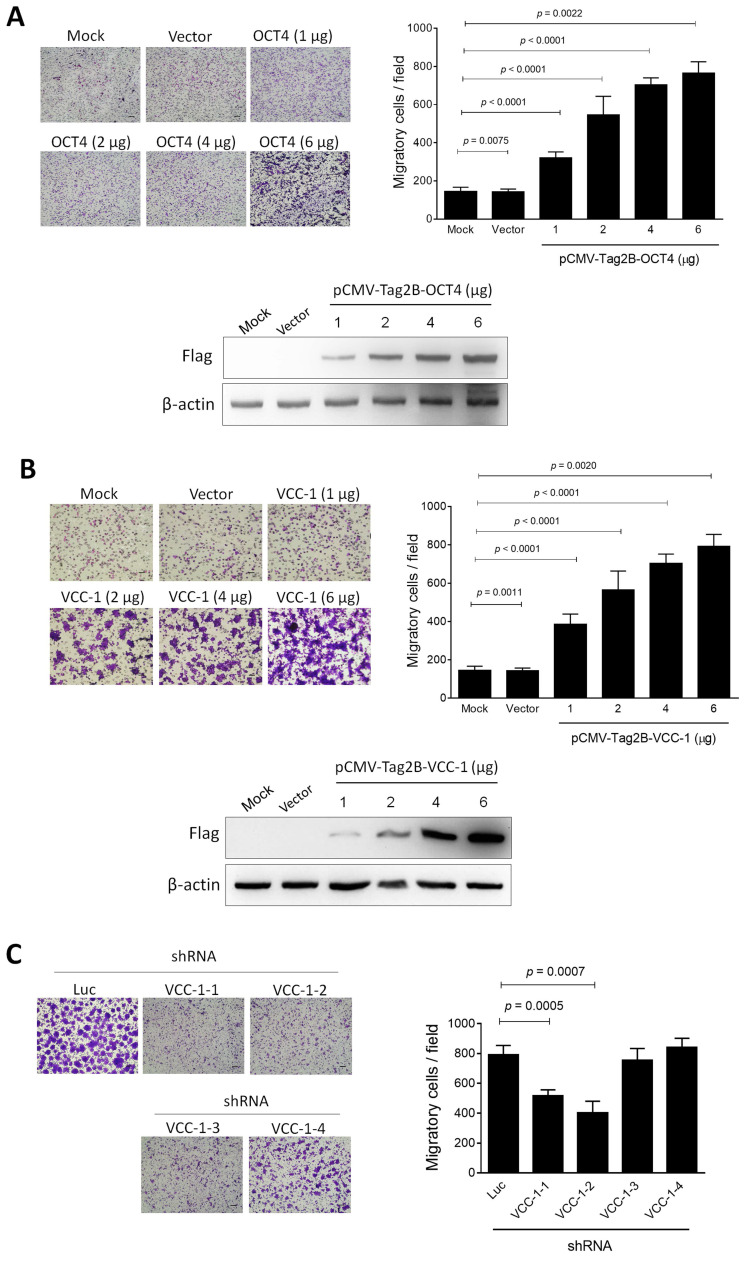
** Lung cancer cells overexpressing OCT4 or VCC-1 attract the migration of macrophage-like THP-1 cells.** (**A, B**) H1299 cells plated in the lower wells in the Boyden chambers were transfected with pCMV-Tag2B (Vector, 6 µg), pCMV-Tag2B-OCT4 (OCT4, 1, 2, 4, and 6 µg), pCMV-Tag2B-VCC-1 (VCC-1, 1, 2, 4, and 6 µg), or mock-transfected. Migration of PMA-induced THP-1 cells were detected by the Boyden chamber assay. Cells that migrated through the membrane of the lower surface in the Boyden chamber were stained with Giemsa solution and visualized by light microscopy (upper panels, × 200 magnification, scale bar = 200 μm, n = 4). Dose-dependent overexpression of OCT and VCC-1 in H1299 cells transfected with Flag-tagged OCT4 and VCC-1 expression vectors were verified by immunoblotting with the anti-Flag antibody, respectively (lower panels). (**C**) The conditioned medium from VCC-1- knockdown A549 cells decreases the migratory ability of macrophage-like THP-1 cells. The culture medium collected from A549 cells that had been transduced with lentiviral vectors expressing shRNAs specific to VCC-1 (#1 to #4) or luciferase (Luc) for 48 h was transferred to the lower Boyden chambers (n = 3). PMA-induced THP-1 cells were seeded in the upper chambers, and their migratory ability was analyzed.

Tumor-associated macrophages (TAMs), a specific type of macrophages, play an important role in the formation of the tumor microenvironment 26. TAMs represent one of the main tumor-infiltrating immune cell types to promote tumor growth, invasion, metastasis, and drug resistance 27, 28. VCC-1 is a chemotactic factor for macrophages and may function as a chemokine that facilitates tumor progression. Nevertheless, the function of VCC-1 in human lung cancer remains unclear. We therefore investigated whether OCT4 and VCC-1, when overexpressed in lung cancer cells, could serve as chemotactic agents to recruit macrophages within the tumor microenvironment. H1299 cells were transfected with pCMV-Tag2B-OCT4 and pCMV-Tag2B-VCC-1, and the conditioned medium was collected after 48 h, respectively. Subsequently, PMA-treated THP-1 cells exhibited a macrophage-like phenotype and demonstrated a significant attraction to the conditioned medium from H1299 cells overexpressing OCT4 or VCC-1. The number of migratory cells was markedly increased in direct correlation with the levels of OCT4 (Figure 4A) or VCC-1 (Figure 4B). Conversely, introduction of effective VCC-1 shRNAs (VCC-1-1 and VCC-1-2) but not ineffective shRNAs (VCC-1-3 and VCC-1-4) into A549 cells to silence VCC-1 expression (Figure 2E) resulted in a subsequent decrease in the migration of macrophage-like THP-1 cells (Figure 4C). Taken together, VCC-1 overexpression and knockdown results in lung cancer cells underscore the pivotal role of VCC-1 in orchestrating the recruitment of macrophages to the tumor microenvironment.

### Knockdown of VCC-1 in human A549 lung cancer cells decreases tumor growth in a human tumor xenograft model

**Figure 5:**
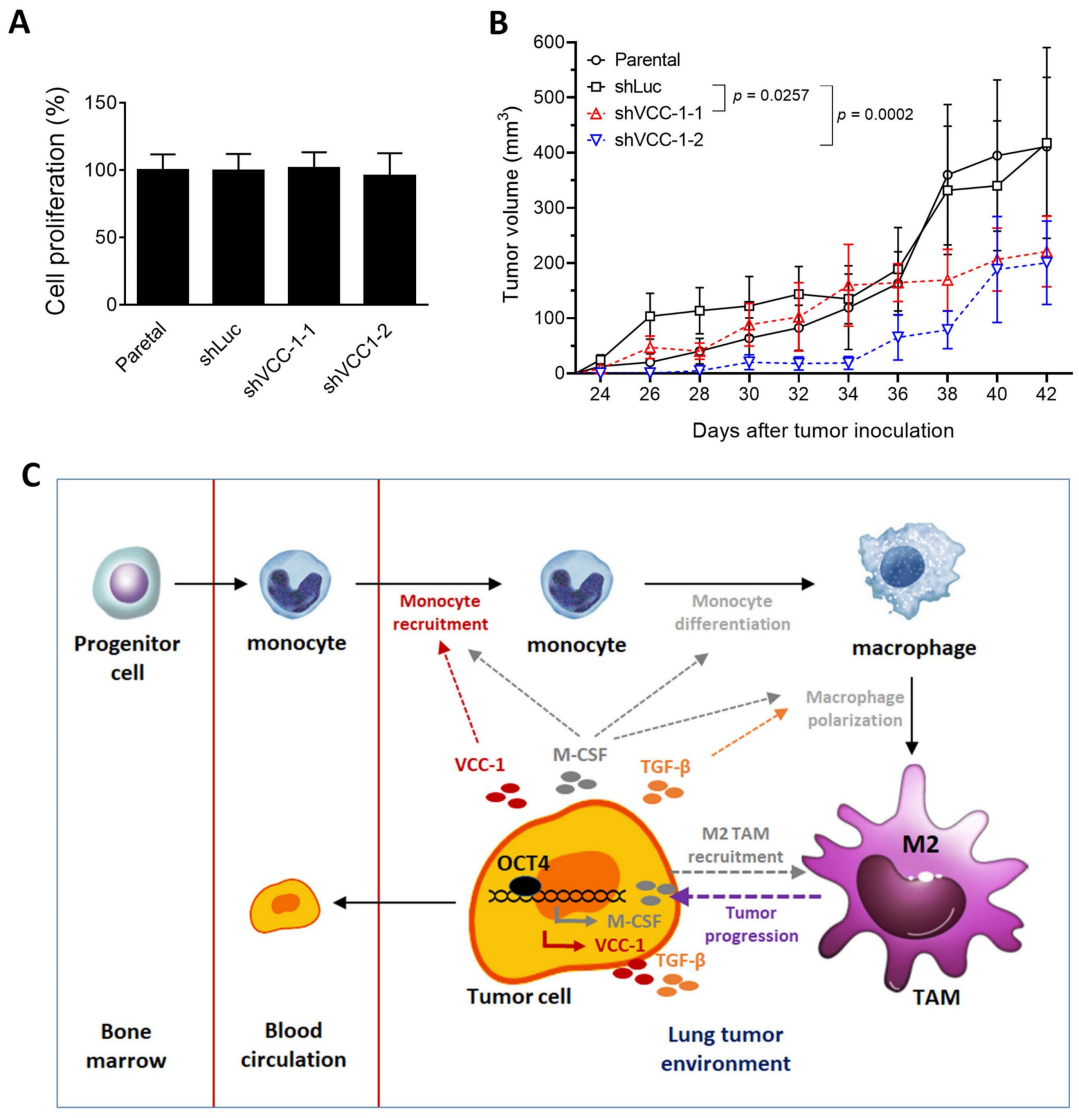
** Knockdown of VCC-1 in A549 lung cancer cells decreases tumor growth in a human tumor xenograft model.** (**A**) Cell proliferative assay of VCC-1-knockdown (shVCC-1-1 or shVCC-1-2), shRNA control (shLuc), and parental A549 cells (n = 4). (**B**) Tumor volumes of mice bearing VCC-1-knockdown (shVCC-1-1 or shVCC-1-2) or control A549 tumors. Groups of four NOD/SCID mice were subcutaneously inoculated with 1 × 10^6^ cells of VCC-1-knockdown or control A549 cells. Tumor volumes of the mice were monitored and measured to elucidate the influence of VCC-1 on tumor development. (**C**) A schematic representation of the OCT4-VCC-1 axis involved in lung cancer progression. OCT4 overexpression in lung cancer cells upregulates VCC-1, which drives tumor aggressiveness through TGF-β secretion and tumor-associated macrophage (TAM) recruitment. OCT4 overexpression in lung cancer cells also promotes M2 macrophage polarization by increasing macrophage colony-stimulating factor (M-CSF) production and enhancing tumor migration, growth, and metastasis. The impact of OCT4 on the upregulation of M-CSF (the pathways in gray color) has been described in our previous paper 4.

Given that OCT4 can upregulate VCC-1 expression, thereby increasing TGF-β and attracting macrophage migration, which may promote tumor progression *in vitro*, we further investigated whether knockdown of VCC-1 expression could decrease tumor growth *in vivo*. To exclude the possibility that knockdown of VCC-1 also impacted cancer cell proliferation and thereby tumor growth, we compared cell proliferation of VCC-1 knockdown and parental A549 cells. Figure 5A shows that knockdown of VCC-1 had no impact on cell proliferation *in vitro*. To corroborate our *in vitro* findings (Figure 3 and 4), we used a xenograft mouse model comprising NOD/SCID mice inoculated with VCC-1-knockdown A549 cells or vector control cells. A549 tumors that develop subcutaneously in NOD/SCID mice have the capacity to metastasize spontaneously and grow on-site in the lung tissue 29, 30. Figure 5B shows that mice bearing A549/shVCC-1-1 or -2 tumors had significantly smaller tumor volumes compared with those bearing A549/shLuc tumors. These results indicate a significant correlation between VCC-1 expression levels and tumor sizes in the A549 tumor model of lung cancer. In addition to the inhibition of OCT4, suppression of VCC-1 expression may also be a potential therapeutic strategy for lung cancer.

## Discussion

In the present study, using clinical samples, overexpression and knockdown approaches in lung cancer cell lines, and mouse tumor models, we identified a molecular mechanism involving the OCT4-VCC-1 signaling axis in lung cancer (Figure 5C). This study underscores the critical role of OCT4 and VCC-1 in lung cancer and offers insights into novel diagnostic and treatment strategies for lung cancer.

The development of tumors shares similarities with the process of embryonic cell development. Genes that regulate embryonic cells also influence tumor formation and progression. OCT4, a crucial regulator of embryonic stem cell regeneration and differentiation, has been demonstrated to possess the capacity to revert differentiated adult cells into induced pluripotent stem cells (iPS), underscoring its significance in both developmental biology and cancer research. 31, 32. The expression of OCT4 has been identified across various cancer types, with evidence suggesting its involvement in promoting the progression of germ cell tumors and its elevated presence in breast cancer cells when compared to normal breast tissue 33, 34. Similar patterns of high OCT4 expression correlating with increased cancer severity have been observed in bladder cancer and non-small cell lung cancer, particularly in regions of lung adenocarcinoma 35, 36. Our research further supports these findings through immunostaining and immunoblot analysis of OCT4 in lung cancer cell lines, demonstrating high expression of OCT4 across most cancers and its role in promoting tumor cell growth and exacerbating the disease. However, the specific molecular mechanisms by which OCT4 contributes to cancerous transformation and regulates downstream genes have not been fully studied. As a transcription factor, OCT4 influences the expression of numerous genes, thereby regulating cell growth, development, and cytokine secretion through various signaling pathways, including STAT3 37. In the present study, our results demonstrate a positive correlation between OCT4 and VCC-1 expression in lung cancer cells, with OCT4 binding to the *VCC-1* promoter to influence its expression.

In the current study, our findings underscore the nuanced and context-specific functions of VCC-1 in tumor progression. While knockdown of VCC-1 in A549 lung cancer cells has no effects on cell proliferation *in vitro* (Figure 5A), it reduces tumor growth in immunodeficient NOD/SCID mice (Figure 5B). Our finding is in line with a recent study demonstrating that CXCL17 (VCC-1) overexpression has no effect on the proliferation of A549 cells 38. However, this differs from prior findings in breast cancer 39, where VCC-1 knockdown reduced proliferation of breast cancer cells *in vitro*. These results suggest a cancer-specific role for VCC-1. In the present study, we therefore proposed that the observed tumor regression in the A549 lung tumor model is primarily mediated through alterations in the tumor microenvironment, particularly the reduction of M2 TAMs and TGF-β secretion. Our results suggest that the capacity of VCC-1 to stimulate TGF-β production is likely to facilitate the recruitment and differentiation of M2 macrophages, which is consistent with its role in promoting immune cell-driven tumor aggressiveness. While the precise mechanisms underlying TAM modulation by VCC-1 remain to be fully elucidated, our data highlight the importance of the OCT4-VCC-1 axis in driving lung cancer progression. Further studies are needed to directly quantify M2 TAMs in VCC-1-knockdown tumor models and explore the broader implications of VCC-1 across different cancer types. These findings contribute to our understanding of the complex interplay between cancer cells and the tumor microenvironment, offering potential avenues for therapeutic intervention.

High VCC-1 expression leads to increased TGF-β secretion by lung cancer cells, supporting its role in suppressing immune responses and facilitating cancer invasion and metastasis 15, 40. This effect, coupled with the attraction of macrophages to the tumor area, suggests the contribution of VCC-1 to creating a tumor-favorable microenvironment. Apart from significant increases in migratory and invasive abilities of VCC-1-overexpressing A549 lung cancer cells 38, our results show that VCC-1 can attract macrophages, suggesting its specificity in immune cell attraction. However, the potential of OCT4-induced VCC-1 expression in lung cancer to attract macrophages warrants further exploration. Additionally, while VCC-1 has been associated with tumor angiogenesis and metastasis in clinical tissues of breast and colorectal cancer 15, our data did not demonstrate significant changes in VEGF secretion by lung cancer cells overexpressing OCT4, suggesting alternative mechanisms of VCC-1 involvement in carcinogenesis. These observations indicate that the regulation of VEGF by VCC-1 may be context-dependent, influenced by specific tumor microenvironments or cancer types. Moreover, although VCC-1 can stimulate VEGF secretion in endothelial cells 15, our study focused on the autocrine effect of VCC-1 within cancer cells, particularly its function in the recruitment of macrophages and the enhancement of TGF-β secretion. Our findings indicate that high VCC-1 expression not only increases macrophage migration to the tumor site but also promotes higher TGF-β secretion by cancer cells. These results indicate that high OCT4 expression in lung cancer cells, followed by increased VCC-1 expression, enhances TGF-β secretion and attracts macrophages, suggesting the role of VCC-1 in creating a tumor-favorable microenvironment or suppressing the immune response in the tumor site. This pathway represents an alternative mechanism through which VCC-1 contributes to tumor progression, independent of VEGF-driven angiogenesis. While further investigation is warranted into the potential paracrine effect of VCC-1 on endothelial cells, our results underscore its broader impact on the tumor microenvironment, emphasizing its pivotal role in driving macrophage-mediated tumor aggressiveness. More importantly, our A549 mouse model experiment demonstrates that knockdown of VCC-1 expression in lung cancer cells decreases tumor size, underscoring the significant correlation between VCC-1 expression and cancer progression. Our findings highlight the high expression of OCT4 in lung cancer cells and its regulatory effect on VCC-1 expression, with the pattern of VCC-1 expression serving as a potential clinical diagnostic marker for lung cancer occurrence and prognosis. The study suggests that further research is required to elucidate the mechanisms by which VCC-1 regulates TGF-β secretion and its overall role in lung cancer.

The present study provides evidence that OCT4 positively regulates VCC-1 expression in lung cancer, contributing to increased TGF-β production and macrophage recruitment, both of which are crucial for tumor progression. The precise mechanistic links between VCC-1 and TGF-β signaling in lung cancer remain to be fully elucidated. However, given that VCC-1 (CXCL17), which has a Glu-Leu-Arg (ELR) motif in the NH2-terminus, belongs to ELR+ CXC chemokine family 41, it may activate TGF-β signaling like other ELR+ family members, which are known to stimulate TGF-β and related immunosuppressive pathways via the CXC chemokine receptors CXCR1 and CXCR2 42, 43. Furthermore, ELR+ CXCLs, which are the ligands for CXCR1 and/or CXCR2, can activate a multitude of downstream pathways, including MAPK, PI3K/AKT, β-catenin, STAT3, and NF-κB 44-48. These pathways collectively contribute to oncogenic processes, such as cell proliferation, metastasis, and angiogenesis. It is noteworthy that VCC-1 binding to CXCR8 has been demonstrated to activate the cAMP pathway and subsequently the ERK1/2 and p38 MAPK pathways via GPCR signaling 49, 50. These results suggest a potential role for VCC-1 in modulating multiple pro-tumorigenic pathways within the tumor microenvironment. Our findings underscore the significance of the OCT4-VCC-1 axis in lung cancer progression, suggesting that targeting this pathway may offer a promising avenue for therapeutic intervention. Further research is necessary to fully elucidate the range of downstream pathways and molecular interactions regulated by VCC-1 in lung cancer.

The apparent lack of correlation between OCT4 and VCC-1 expression in the CL1 cell lines (Figure 1E) underscores the intricate nature of VCC-1 regulation and indicates that OCT4 may not be the sole upstream regulator in these particular cell lines. Other transcriptional or post-transcriptional mechanisms may contribute to VCC-1 expression. Further studies are required to identify these regulatory factors. Furthermore, the phenotypic drift observed in long-term *in vitro* cell culture may also account for reduced OCT4 expression in CL1 cell lines, potentially affecting its regulatory role. Nevertheless, our examination of clinical human lung adenocarcinoma specimens reveals a notable positive correlation between OCT4 and VCC-1 expression (Figure 1D), thereby substantiating the pivotal role of the OCT4-VCC-1 axis in lung cancer progression. These findings highlight the necessity of considering both *in vitro* and *in vivo* models to fully elucidate the regulatory networks of VCC-1 and their implications in tumor biology.

OCT4 plays a crucial role in maintaining the self-renewal of undifferentiated embryonic stem cells. Its expression is associated with the ability of cancer cells to proliferate, resist conventional therapies, and metastasize. In the current study, we illustrate the role of the OCT4-VCC-1 axis in lung cancer. Treatments for lung cancer, especially those targeting specific markers, often involve a combination of strategies, including targeted therapy, chemotherapy, radiation therapy, and immunotherapy. Some treatments, including retinoic acid that targets OCT4, have been proposed 51. However, treatments specifically targeting OCT4 would fall into the category of targeted therapy for a single treatment. Cancer cells with high OCT4 expression can potentially manipulate the immune system to alter tumor immune evasion 52. To enhance the tumor-specific targeting and avoid side effects caused by upstream molecules or pathways, VCC-1 might be a better choice for dealing with the OCT4/VCC-1 axis. Furthermore, miR-325-3p that target VCC-1 exerts anticancer activities 53. Given the complexity and rapid evolution of cancer treatments, especially in the realm of targeted therapies and precision medicine, there may be new developments and clinical trials focusing on the OCT4/VCC-1 axis in lung cancer. Therefore, our findings highlight the importance of the OCT4-VCC-1 signaling pathway in lung cancer progression. In addition, the OCT4-VCC-1 axis may be a potential therapeutic target for lung cancer.

## Supplementary Material

Supplementary table.
