# Supplementary material for: OCT4 promotes lung cancer progression through upregulation of VEGF-correlated chemokine-1
Source: Int J Med Sci. 2025 Jan 13;22(3):680–95. doi: 10.7150/ijms.102505 (PMC11783078; doi:10.7150/ijms.102505)
Supplement: Supplementary file 1 — Supplementary table. [file ijmsv22p0680s1.pdf]

Supplementary Table | A list of reagents and resources used in the study

Reagent or resource	Source	Identifier
**Antibodies**		
Rabbit polyclonal anti-VCC-1 (IHC: 1:100) (WB: 1:1000)	Proteintech	Cat# 18108-1-AP; RRID: AB_2878502
Mouse monoclonal anti-OCT4 (IHC: 1:100) (WB: 1:1000)	Genetex	Cat# GTX627419; RRID: AB_11176459
Mouse monoclonal Anti-β-Actin−Peroxidase (1:20000)	Sigma-Aldrich	Cat# A3854; RRID: AB_262011
Rabbit polyclonal anti-Flag (DYKDDDDK Tag D6W5B) (WB: 1:3000)	Cell Signaling Technology	Cat#; 14793; RRID: AB_2572291
Mouse monoclonal anti-OCT4 (C-10) (ChIP: 1:100)	Santa Cruz	Cat# sc-5279; RRID: AB_628051
Normal Mouse IgG	Cell Signaling Technology	Cat# 2729; RRID: AB_1031062
Peroxidase AffiniPure Goat Anti-Mouse IgG (H+L) (1:5000 to 1:10000)	Jackson ImmnunoResearch	Cat#115-035-003 PRID: AB_11015289
Peroxidase AffiniPure Goat Anti-Rabbit IgG (H+L) (1:5000 to 1:10000)	Jackson ImmnunoResearch	Cat#115-035-114 PRID: AB_11015289
**Biological Samples**		
Human lung cancer specimens	This paper	N/A
NHRI-BC-008 primary breast cancer cells (TNBC)	This paper	N/A
**Chemicals, Peptides, and Recombinant Proteins**		
phorbol 12-myristate 13-acetate (PMA)	Merckmillipore	Cat#PB-139
Recombinant Human IL-4 Protein	R&D Systems	Cat#204-IL
Recombinant Human CXCL17/VCC-1 Protein	R&D Systems	Cat#4027-DM
3-amino-9-ethylcarbazole	Thermo Fisher Scientific	Cat#147870050
gelatin	Sigma-Aldrich	Cat#1288485
**Commercial Assays**		
yT&A cloning vector kit	Yeastern Biotech	N/A
PrimeScript™ High Fidelity RT-PCR Kit	Clontech/Takara Bio	Cat#RR022
Immobilon Western Chemiluminescent HRP Substrate	Merckmillipore	Cat# WBKLS0500
QuickGene RNA Cultured Cell Kit	AUTOGEN	Cat# FK-RCS2
PrimeScript™ RT Reagent Kit	Clontech/Takara Bio	Cat#RR037
SYBR premix Ex Taq kit	Clontech/Takara Bio	Cat#RR420
Dual-Luciferase® Reporter Assay System	Promega	Cat#E1910
EZ-ChIP™ kit	Merckmillipore	Cat#17-371
Human TGF-beta 1 DuoSet ELISA	R&D Systems	Cat#DY240
Human VEGF DuoSet ELISA	R&D Systems	Cat#DY293B

Cell Counting Kit-8 (CCK-8)	Dojindo Laboratories	Cat#CK04
**Experimental Models: Cell Lines**		
Human embryonic lung cells (HEL299)	Bioresource Collection and Research Center, Taiwan	N/A
Human non-small cell lung carcinoma cells (H1299)	Bioresource Collection and Research Center, Taiwan	N/A
Human lung adenocarcinoma epithelial cells (A549)	Bioresource Collection and Research Center, Taiwan	N/A
Human acute monocytic leukemia cells (THP-1)	Bioresource Collection and Research Center, Taiwan	N/A
Human: HEK-293T line	Bioresource Collection and Research Center, Taiwan	N/A
A panel of human lung adenocarcinoma cell lines (CL1-0, CL1-1, CL1-3, CL1-5, and CL1-5F4)	Laboratory of Pan-Chyr Yang, Taiwan	N/A
**Experimental Models: Organisms/Strains**		
Mouse: NOD/SCID mice	NCKU animal center	N/A
**Oligonucleotides**		
Primer: *OCT4*-specific primer Forward: 5’-GTCCGAGTGTGGTTCTGTA -3’	This paper	N/A
Primer: OCT4-specific primer Reverse: 5’-CTCAAGTTTGAATGCATGGGA -3’	This paper	N/A
Primer: VCC-1-specific primer Forward: 5’-ATGAAAGTTCTAATCTCTTCCCTC -3’	This paper	N/A
Primer: VCC-1-specific primer Reverse: 5’-CTACAAAGGCAGAGGCAAAGCTTC -3’	This paper	N/A
Primer: OCT4-qPCR primer Forward: 5’-CCTGAAGCAGAAGAGGATCACC -3’	This paper	N/A
Primer: OCT4-qPCR primer Reverse: 5’-AAAGCGGCAGATGGTCGTTTGG -3’	This paper	N/A
Primer: VCC-1-qPCR primer Forward: 5’-ACAGTGTCTGGGCTGCCAAAGA-3’	This paper	N/A
Primer: VCC-1-qPCR primer Reverse: 5’-GGCTCTGGAATGCTTGTTTGGC-3’	This paper	N/A
Primer: GAPDH-qPCR primer Forward: 5’-ACTTCAACAGCGACACCCACT-3’	This paper	N/A
Primer: GAPDH-qPCR primer Reverse: 5’-GCCAAATTCGTTGTCATACCAG-3’	This paper	N/A

Primer: VCC-1- promoter primer Forward: 5’-TGACACAAATAATGTTCTTGAGA-3’	This paper	N/A
Primer: VCC-1- promoter primer Reverse: 5’-GCTTTAGTCCCAGGCCAGCGTTC-3’	This paper	N/A
Primer: VCC-1-ChIP primer Forward: 5’-TTTCTGCCTACAGGTTCCACT	This paper	N/A
Primer: VCC-1-ChIP primer Reverse: 5’-CCTCAATTCTGATCTGCTCACTT	This paper	N/A
**Lentivirus shRNA**		
Target gene: *OCT4*	Academia Sinica, Taiwan	TRCN0000004880
Target gene: *OCT4*	Academia Sinica, Taiwan	TRCN0000004883
Target gene: *VCC-1*	Academia Sinica, Taiwan	TRCN0000134061
Target gene: *VCC-1*	Academia Sinica, Taiwan	TRCN0000138165
Target gene: *VCC-1*	Academia Sinica, Taiwan	TRCN0000137946
Target gene: *VCC-1*	Academia Sinica, Taiwan	TRCN0000136414
Target gene: *VCC-1*	Academia Sinica, Taiwan	TRCN0000136148
Scamble-target control: Luciferase	Academia Sinica, Taiwan	TRCN0000072246
**Recombinant DNA**		
pSin-EF2-OCT4-Pur	Laboratory of James Thomson, UW–Madison Stem Cell and Regenerative Medicine Center, Madison, WI, USA	Addgene plasmid #16579
pSin-EF2-GFP-Pur	This paper	N/A
pCMV-tag2B	Stratagene	Catalog #211172
pCMV-tag2B-hOCT4	This paper	N/A
pCMV-tag2B-hVCC-1	This paper	N/A
pLKO.1-puro-based lentiviral vectors	Academia Sinica, Taiwan	N/A
pMD2.G	Laboratory of Didier Trono, École Polytechnique Fédérale De Lausanne, Switzerland	Addgene plasmid #12259

psPAX2	Laboratory of Didier Trono, École Polytechnique Fédérale De Lausanne, Switzerland	Addgene plasmid #12260
pFRL2-hVCC-1p-Luc	This paper	N/A
**Software and Algorithms**		
GraphPad Prism (v.6.01)	GraphPad Software	https://www.graphpad.com/
